# Investigating the interconnectedness of carbon, fossil energy, and financial markets: A dynamic spillover index approach

**DOI:** 10.1371/journal.pone.0295363

**Published:** 2023-12-14

**Authors:** Tianyou Li, Yanbing Ju, Peiwu Dong

**Affiliations:** School of Management and Economics, Beijing Institute of Technology, Beijing, Asia, China; The University of Hong Kong, HONG KONG

## Abstract

Against the background of the global active pursuit of carbon neutrality, this paper uses the DY spillover index method to analyze the spillover network effects between carbon, fossil energy and financial markets. The research results show that the spillover effects between these three markets change over time, with an average spillover index of 25.30%, showing a significant mutual influence. Further analysis found that the EU carbon market plays an important role in spillover effects. Especially under the influence of extreme events, the spillover effects reach their peak. At this time, the degree of mutual influence between markets is as high as 60.01%. In addition, during the COVID-19 epidemic, the spillover effect of the EU carbon market on other markets also reached its maximum, indicating that the epidemic increased the contagion of cross-market risks and caused the carbon market to bear greater risks. The research results of this article have important guiding significance for environmental protection investment and emphasize the importance of formulating differentiated environmental protection policies in different time frames. Facing the dual challenges of global climate change and promoting the goal of carbon neutrality, governments and relevant institutions should pay close attention to changes in spillover effects between markets and timely adjust environmental protection policies to achieve maximum results.

## 1. Introduction

As the global climate change problem becomes increasingly severe, the carbon emissions trading market, as an innovative environmental protection method, promotes emission reduction and promotes low-carbon development through a market-oriented approach. However, the carbon emissions trading market does not exist in isolation, and its operation and development are affected by many factors. As a basic input to economic activities in today’s industrialized world, fossil energy combustion is the main source of carbon emissions, with 73.2% of global greenhouse gas emissions coming from fossil fuel consumption [1]. Fossil fuels cause higher net greenhouse gas emissions than renewable energy sources. The development of the carbon market is inseparable from the development of fossil fuels. The economic fundamentals hypothesis believes that fundamentals can lead to cross-market price correlations [2, 3]. The price of traditional fossil fuels can affect carbon emissions through the market supply and demand of energy, thereby affecting carbon emissions. For example, when fossil fuel prices fall, the demand for energy increases, leading to an increase in carbon emissions, which in turn causes the price of carbon allowances to rise [4]. The EU’s Emissions Trading System (ETS) increases the cost of energy production from fossil fuels, thereby creating carbon price risks [5]. In addition, the carbon market, as a financial commodity, can be traded in the financial market. Therefore, there may be a strong influence between the financial market and the carbon market. Price fluctuations in the carbon market may be passed on to related asset prices in the financial market, such as carbon financial products, energy stocks, etc. [6]. In addition, the supply and demand situation in the EU carbon market may affect the liquidity of financial markets and the trading behavior of market participants. For example, if there is an oversupply of carbon allowances, this could lead to a decrease in the price of carbon allowances, which in turn could lead to a decrease in the value of financial instruments related to carbon allowances. On the other hand, capital flows and investment behavior in the financial market may also affect the supply and demand relationship and price level of the EU carbon market [7]. For example, if financial institutions increase their investment in the carbon market, this may lead to increased demand for carbon allowances, which may in turn lead to an increase in the price of carbon allowances.

Based on the above analysis, this article puts forward the first hypothesis.

a significant linkage between carbon, fossil energy and financial markets.

In addition, the correlations between carbon and energy markets and financial markets may also change over time due to the heterogeneity of different market participants and changing market conditions. For example, when risks arise in the carbon market, they will be transmitted more to the energy market rather than the financial market. This is because different market participants have different understandings and judgments about the risks of the carbon market, and different market participants will also take different measures to deal with risks. For example, when carbon market prices fall, energy companies may reduce their demand for carbon allowances, while financial institutions may continue to hold carbon allowances and wait for their value to return. In addition, due to the different goals and preferences of market participants, the price formation mechanisms between different markets are also different, which may also lead to heterogeneity in market spillover effects [8]. For example, in some cases, price fluctuations in the carbon market may have a greater impact on prices in the energy market, while in other cases, financial market flows and investment behavior may have a greater impact on prices in the carbon market. Impact. Therefore, we propose our second hypothesis.

H2: The spillover effects between carbon, energy markets and financial markets are time-varying and heterogeneous.

In addition, extreme events may also have an impact on the correlation between different markets. For example, fluctuations in energy prices may affect the price and trading volume of the carbon market, and may affect investors’ risk preferences and emotions [9]. Bao Z and Huang D found that, both fintech companies and traditional banks experienced significant changes in the lending environment during the COVID-19 period. By analyzing loan data during this period, found that fintech companies are more likely to expand their credit channels to new, financially strapped borrowers after the outbreak. However, although the loan default rate of fintech companies has tripled after the outbreak of the epidemic, the loan default situation of traditional banks has not changed significantly. These results indicate that although shadow banks have provided loan facilities during crises, these institutions may also become vulnerable when default rates soar. This further proves the impact of extreme events on spillover effects between different markets [10]. After extreme weather events, investors may become more cautious about investing in energy and carbon markets, which may lead to a decrease in trading volume and liquidity in the market, thereby affecting risk assessment and investment decisions for energy-related assets. During extreme events, some market participants may suspend trading or reduce trading volume, which may lead to a decrease in market liquidity. This change in liquidity may affect the spillover effects between different markets. For example, the correlation between the carbon market and the financial market may be weakened due to the decline in liquidity [11]. Extreme events may not only affect market liquidity, but also affect the market’s price formation mechanism. After extreme events, some investors may be more focused on short-term market dynamics and unwilling to make long-term investments. This may result in greater price volatility in the market, thereby increasing investor risk. In addition, extreme events may also affect investors’ risk preferences and sentiments, thereby affecting market price formation. Therefore, this paper proposes the third hypothesis:

H3: Extreme events will affect the spillover effects between carbon markets, energy markets and financial markets.

explore the spillover effects between carbon, fossil energy and financial market markets using the spillover effect framework constructed by Diebold and Y1 lmaz (2014). Our study makes an important contribution to the literature. First, most empirical studies use traditional time-domain techniques to analyze the connection between carbon and energy markets and focus on carbon and traditional energy markets [12–14]. The relationship between carbon markets and financial markets remains largely unstudied. Secondly, in order to test whether the COVID-19 epidemic has affected the dynamic spillovers between carbon and energy markets, we use marginal net risk spillover analysis and use complex network models to comprehensively identify risk contagion characteristics and propagation paths. The research results provide a basis for formulating risk prevention policies and reasonable carbon trading policies under extreme risk event scenarios, and provide support for the demonstration of carbon financing to promote carbon emission reduction strategies. The remainder of this article is organized as follows. Section 2 provides a literature review. Section 3 introduces the model approach. Section 4 discusses the data and results. Section 5 summarizes the main findings and proposes some meaningful policy recommendations.

## 2. Literature review

Many studies document the relationship between fossil energy and carbon markets. For example, Wen et al. (2017) explored the dependence between EUAs and energy-related commodity futures prices and found that coal plays a relatively important role in reducing carbon risks [15]. Liu et al. (2023) studied the volatility spillover and dynamic correlation between EUA and fossil energy prices and found limited evidence of volatility spillover between EU ETS and fossil energy markets [14]. Qiao et al. (2023) used time-varying structured vector stochastic fluctuation autoregression (TVP-VAR-SV) to analyze the time-varying correlation between carbon and fossil energy futures markets and found that the carbon market is highly susceptible to changes in the coal market [12]. Liu et al. (2023) used quantile regression to analyze the marginal effect of energy prices on carbon price changes and found that this effect is asymmetric and negative. Existing research focuses on fossil energy (i.e., crude oil, coal, and natural gas prices) and CET markets [13]. In fact, there is a configuration effect in the transmission path from the fossil energy market to the carbon market [16]. Fluctuations in energy commodity prices will cause adjustments in energy consumption behavior, leading to changes in total carbon emissions, which will in turn affect CET prices.

With the rapid growth of the financial market sector, the connection between financial markets and carbon markets is becoming increasingly close. Carbon prices synchronize with macroeconomic fluctuations and show the procyclicality of the economy [17] However, the relationship between financial economic markets and carbon markets remains unclear, but there is no doubt that there is a strong link between stock markets and EUA prices. On the one hand, the stock market can reflect the economic conditions of an economy. Positive economic conditions are expected to improve company profits, which makes company stocks more attractive as dividends to shareholders are expected to be larger [18]. On the other hand, higher economic activity leads to higher energy demand, which leads to higher carbon emissions, thus giving EUA price increases [19]. Therefore, the causal relationship between stock markets and EUA prices appears to run from the former to the latter. Still, EUA prices may change the economic incentives of manufacturing companies, and this change may be priced in the stock market. Therefore, it is necessary to examine the causal relationship between EUA prices and stock market indexes.

Furthermore, in addition, Bao and Huang (2021) analyzed the connectivity and information efficiency of the Shanghai Stock Exchange A-share market in different periods, including calm periods and high leverage periods, and used Pearson correlation, maximum strongly connected subgraphs, and 3 σ Principle to dynamically determine the threshold for constructing correlation in the Shanghai Stock Exchange A-share market. Research has found that the internal connectivity of the finance, energy, and utility sectors is stronger than other sectors [20]. Chen et al. (2021) divided the period from 2005 to 2018 into eight bull and bear market stages based on the daily stock returns of A-shares on the Shanghai Stock Exchange (SSE) to examine the interactive patterns of China’s financial market, using the Least Absolute Shrinkage and Selection Operation (LASSO) method to construct a stock network and compare the heterogeneity of bull and bear markets. The empirical results show that the connection effect during bear markets is more significant than during bull markets, leading to abnormal volatility in the stock market [21]. Bao Z and Huang D proposed a Time Zone Vector Autoregressive (VAR) model to study the synchronization of global financial markets. By analyzing daily data from stock markets in 36 countries, static and rolling window methods were used to analyze the subprime mortgage, European debt crisis, and COVID-19 crisis. Studying the VAR coefficient reveals resonance effects in global systems. The results of density and volatility studies indicate the existence of conduction mechanisms and abnormal structural changes. The strength analysis reveals the mechanism of information transmission across continents and emphasizes the unique role of specific stock markets [22]. In general, From a model approach perspective, the causal dynamics of prices in financial markets, energy and carbon markets are studied using various data sets and econometric models such as dynamic conditional correlation (DCC) multivariate generalized autoregressive conditional heteroskedasticity (GARCH) model [23] nonlinear autoregressive distributed lag model [24], Copula model [25, 26], quantile regression method [27], TVP-VAR-SV [28, 29] and Diebold and Yilmaz (DY) dynamic connectivity method [30, 31]. However, these traditional econometric methods cannot reveal the possible spillover effects of energy and carbon markets in different frequency ranges. Although Jiang and Ma (2021) used asymmetric BEKK- and DCC-GARCH models to study the volatility spillover and dynamic correlation between carbon, fossil energy and financial market markets from a multi-scale perspective, they did not establish unified network system framework Internal quantities capture the intensity and magnitude of spillover [32]. In addition, the role of artificial intelligence in processing this data and predicting future trends can also be considered [33–35].

Based on the existing literature, financial measurement methods to examine the relationship or spillover effects between the carbon market and other markets generally include: VAR model, multivariate GARCH family model, DY spillover index model, quantile regression model, Copula model, CoVaR model, etc. Among them, the VAR model and the multivariate GARCH family model focus on describing the relationship between returns or volatility among markets. In models targeting extreme risk spillover effects, the quantile regression model can only measure the static linear correlation between quantiles among variables, the Copula model is limited by the specific form of the Copula function and there is a certain degree of subjectivity in the modeling process, while the CoVaR model is mainly used to characterize the tail risk spillover effects between binary variables, and it is difficult to measure the extreme risk among multiple variables. Systemic spillover levels. Judging from the research content, the existing literature mostly focuses on the traditional energy market and carbon market, and few scholars link the traditional energy market with the carbon market and financial market. In view of the limitations of existing research, this paper uses D Y overflow to characterize the relationship between the three. The advantage of this model is that it not only considers the intensity of the overflow between the three, but also reveals the degree, amplitude and direction of the overflow in the connection network. Furthermore, the heterogeneity of the impact of traditional energy markets and financial markets on EUA is examined. Finally, the impact of extreme events on system linkage is depicted.

## 3. Materials and methods

This paper uses the Diebold and Yilmaz (DY) spillover index [19] for empirical research. The model is based on a time-varying variance-covariance structure that allows capturing possible changes in the underlying structure of the data in a more flexible and robust manner. Since heteroscedastic processes are usually better than homoscedastic processes, the time-varying variance—covariance structure is beneficial to the model to produce regression results that are more consistent with economic reality. Specifically, this article first defines an n- order VAR model for the input time series *Y*_*t*_:

$$Y_{t}=\sum_{k=1}^{K}\varphi_{k}Y_{t-1}+\varepsilon_{t}$$

Among them: *Y*_*t*_ = (*Y*_1*t*_,*Y*_2*t*_,…,*Y*_*Nt*_). ε_*t*_ ∈ (0, ∑)is a vector of independent and identically distributed disturbances. In this paper, *Y*_*t*_ represents the time series of financial markets, carbon markets, and energy markets. The same expression using a moving average is:

$$Y_{t}=\sum_{k=0}^{\infty }A_{k}\varepsilon_{t-1}+\varepsilon_{t}$$

The *N* × *N* coefficient matrix *A*_*i*_ obeys recursion $A_{i}=\Phi_{1}A_{i-1}+\Phi_{2}A_{i-2}+\cdots +\Phi_{p}A_{i-p}$, where *A*_0_ is *N* × *N* the identity matrix, and *i* when it is less than 0, *A*_*i*_ = 0. After constructing the spillover effects and eliminating the possible dependence of the results on the order, variance decomposition was performed. Variance decomposition allows an assessment of the proportion of the forecast error variance that is due to a shock *Y*_*j*_ for each *i* ≠ *j* forecast *Y*_*i*_.

By utilizing the generalized VAR framework, the problem that variance decomposition depends on the order of variables is circumvented. This framework produces an ordering-invariant variance decomposition that does not attempt to orthogonalize shocks but instead allows for correlated shocks but accounts for them appropriately using historically observed error distributions. Since the shocks to each variable are not orthogonalized, the contributions to the forecast error variance do not necessarily sum to unity. So, when *i*,*j* = 1,2,⋯,*N*, and *i* ≠ *j*, is defined to represent $\theta_{ij}^{g}(H)$the step prediction error variance decomposition *H* = 1,2,⋯of KPPS, for *H*, there is:

$$\theta_{ij}^{g}(H)=\frac{\sigma_{ii}^{-1}\sum_{h=0}^{H-1}{\left(e_{i}^{\prime }A_{h}\sum e_{j}\right)}^{2}}{\sum_{h=0}^{H-1}\left(e_{i}^{\prime }A_{h}\sum A_{h}^{\prime }e_{j}\right)}$$

where is ∑ the variance matrix of σ_*ii*_ the error vector, ε is *i* the standard deviation of the error term of the equation, *e*_*i*_ is *i* the selection vector whose element is 1, and the others are 0. As mentioned above, the sum of the elements in each row of the variance decomposition table is not equal to 1, that is $\sum_{j=1}^{N}\theta_{ij}^{g}(H)\neq 1$. In order to use the information available in the variance decomposition matrix when calculating the spillover index, we normalize each entry of the variance decomposition matrix by a row sum:

$${\hat{\theta }}_{ij}^{g}(H)=\frac{\theta_{ij}^{g}(H)}{\sum_{j=1}^{N}\theta_{ij}^{g}(H)}$$

Note that the construction $\sum_{i,j=1}^{N}{\hat{\theta }}_{ij}^{g}(H)=N$and $\sum_{j=1}^{N}{\hat{\theta }}_{ij}^{g}(H)=1.$

Using the volatility contribution from variance decomposition, a total volatility spillover index can be constructed:

$$TSI=S^{g}(H)=\frac{\sum_{i,j=1,i\neq j}^{N}{\hat{\theta }}_{ij}^{g}(H)}{\sum_{i,j=1}^{N}{\hat{\theta }}_{ij}^{g}(H)}=\frac{\sum_{i,j=1,i\neq j}^{N}{\hat{\theta }}_{ij}^{g}(H)}{N}$$

The total spillover index measures the contribution of the spillover effects of category volatility shocks to the total forecast error variance. In this paper, TSI is the overall spillover index between the financial market, carbon market, and energy market.

Net volatility spillover provides summary information on the net contribution of each domain to the volatility of other domains. It is also interesting to examine net volatility for spillovers, which we define as:

$$S_{ij}^{g}(H)=\frac{{\hat{\theta }}_{ij}^{g}(H)}{\sum_{k=1}^{N}{\hat{\theta }}_{ik}^{g}(H)}-\frac{{\hat{\theta }}_{ji}^{g}(H)}{\sum_{k=1}^{N}{\hat{\theta }}_{jk}^{g}(H)}$$

Among them: is $\frac{{\hat{\theta }}_{ij}^{g}(H)}{\sum_{k=1}^{N}{\hat{\theta }}_{ik}^{g}(H)}$the impact $\frac{{\hat{\theta }}_{ji}^{g}(H)}{\sum_{k=1}^{N}{\hat{\theta }}_{jk}^{g}(H)}$transmitted *j* from the market to the total volatility, is the impact *i* transmitted *i* from the market *j* to the total volatility. For example, it can represent volatility spillovers from the financial market to the carbon market. The net pairwise volatility spillover between *i* and the market is simply *j* the difference between the total volatility shock *i* transmitted from the market to *j* and the total volatility shock *j* transmitted from to. *i* Through net volatility spillover, the volatility spillover effect of the independent variable on the dependent variable can be obtained, thereby constructing the risk relationship between variables.

Pairwise directed overflow is defined as:

$$DSI_{i\to j}(H)=\frac{\sum_{i=1,i\neq j}^{N}{\hat{\theta }}_{ij}^{g}(H)}{\sum_{i=1}^{N}\theta_{ij}^{g}(H)}\times 100$$

$$DSI_{i\leftarrow j}(H)=\frac{\sum_{j=1,i\neq j}^{N}{\hat{\theta }}_{ij}^{g}(H)}{\sum_{j=1}^{N}\theta_{ij}^{g}(H)}\times 100$$

The paired spillover index here refers to the intensity of the volatility spillover effect between two markets.

Overall, the advantages of DY spillover index are summarized as follows:

Firstly, the DY spillover index not only considers the direction of spillovers, but also the size of spillovers, which can include carbon, energy, and financial markets in a framework for analysis. Secondly, the DY spillover index can compare the size of spillover effects between different markets, providing valuable reference information for policy makers and investors. By comparing the spillover effects of carbon markets, energy markets, and financial markets, we can better understand the interrelationships and impacts between markets. Thirdly, the DY spillover index is sensitive to small changes in the market and can capture the interactions and impacts between carbon markets, energy markets, and financial markets. This enables the method to reflect market changes and trends in a timely manner.

However, the DY spillover index also has certain limitations.

Firstly, the DY spillover index method relies on a large amount of historical data to calculate spillover effects, and in the face of certain emerging markets or insufficient data, this method may be difficult to obtain accurate results. However, the EU carbon market selected in this article as the research object is one of the largest carbon markets in the world, which has been in operation for more than ten years and has entered the fourth stage since 2021. This means that the EU carbon market is relatively mature and can provide sufficient historical data for analysis. In addition, for financial markets and traditional energy markets, their operating time is longer and more mature than carbon markets. The historical data of these markets is also more abundant, which can better support the calculation and analysis of DY spillover index. Therefore, the problem studied in this article can avoid the limitations of the DY spillover index method caused by insufficient data or immature markets. Secondly, the DY spillover index requires complex calculations and analysis, and may take longer to calculate for large-scale market data. This may limit its application in real-time analysis and decision-making. However, in this study, due to the sample interval we selected from January 1, 2013 to February 10, 2023, after matching the same date data, we ultimately obtained 2242 pieces of data as samples. In addition, a total of 9 indicators were selected for measurement in the financial market, carbon market, and energy market of this article. This data scale is smaller than minute and hour level data, but more abundant than monthly and quarterly data. Therefore, through this compromise approach, we strive to enrich the extractable market information while avoiding overly complex calculation and analysis processes. In this way, the potential drawbacks of the DY overflow index method were successfully avoided in the research question of this article.

## 4. Results and discussion

### 4.1 Data selection

As the world’s largest carbon market, the EU ETS has been operating for more than 10 years and has gradually matured. It has entered the fourth stage starting in 2021, and the third stage has also accumulated rich experience since it was launched in 2013, achieving emission control enterprises Both the total amount and intensity of carbon emissions have declined. Studying the dynamic changes in its price will help to better understand the rules of carbon market operation, and can provide valuable suggestions for the development of my country’s carbon market. Therefore, this study selects the monthly data of EU emission allowance (EUA) futures settlement prices as the main research object of this article. In addition, for the influencing factors of the carbon market, the closing prices of eight indicators in the financial market and energy market were selected. The data were all sourced from the WIND database. The sample interval was selected from January 1, 2013 to February 10, 2023. According to Common data matching, the final sample size is 2242 data, the specific data selection and symbol sources are as shown in Table 1:

**Table 1:** Indicator selection.

Classification	Market	Indicator Name	Symbolic Representation	Unit
**Dependent Variable**	EU carbon market	Futures Settlement Price: EU Emissions Allowances	EUA	Euro/ton CO2 equivalent
**Influencing Factors**	Financial market	United States: S&P 500 Index	SP500	point
		United States: Treasury Yield: 3 Month	T-BILL	%
		spread	BAA-AAA	%
		Paris CAC40 Index	CAC40	point
		London FTSE 100 Index	FTSE100	point
		Frankfurt DAX Index	DAX	point
	energy market	Futures Closing Price: IPE British Gas	IPE	Pennies/sem
		Futures settlement price: Brent crude oil	BRENT	USD/barrel

**Fig 1:**
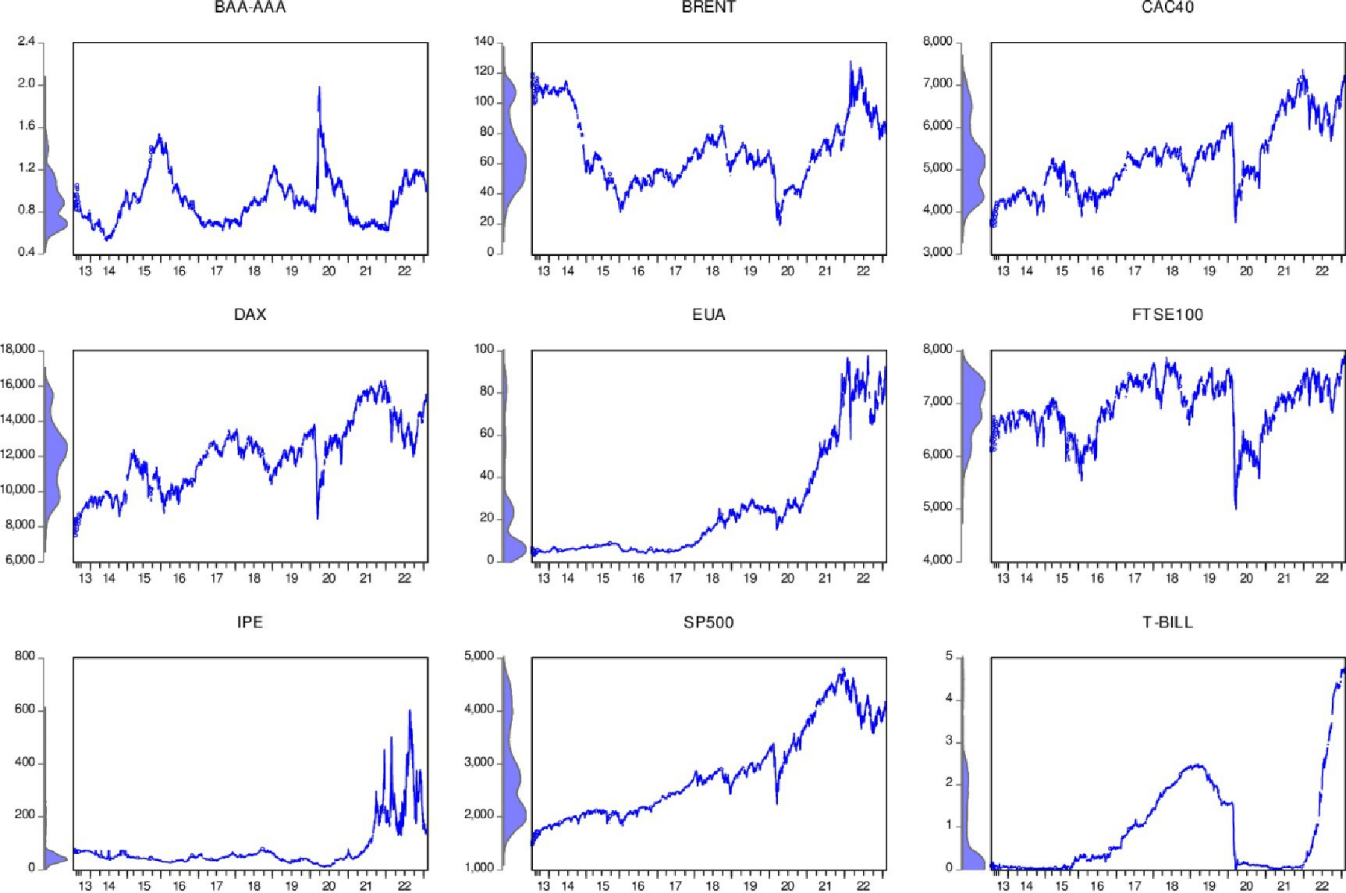
Variable trend and distribution display.

The selected indicator closing prices is shown in Fig 1:

It can be seen from Fig 1 that the closing prices of each indicator show a non-linear trend. For EU carbon market prices, they have shown an overall upward trend since 2013. It can be seen from this that carbon prices have shown an overall upward trend since 2013, and there has been a slight decline in 2020, indicating that the carbon market has also been affected by the new coronavirus epidemic and has shown a downward trend in prices. After that, the epidemic was effectively controlled, and "carbon neutrality" The calls for "harm" and "peak carbon" and the EU carbon market entering the fourth stage in 2021. The superposition of three major events has caused the EU carbon price to rise rapidly, from less than 40 euros/carbon dioxide equivalent at the end of 2020 to a maximum of 90.81 Euro/CO2 shows that the current international market pays more attention to emission reduction and effectively reduces carbon emissions through market means, thereby completing the dual carbon goals as planned.

**Fig 2:**
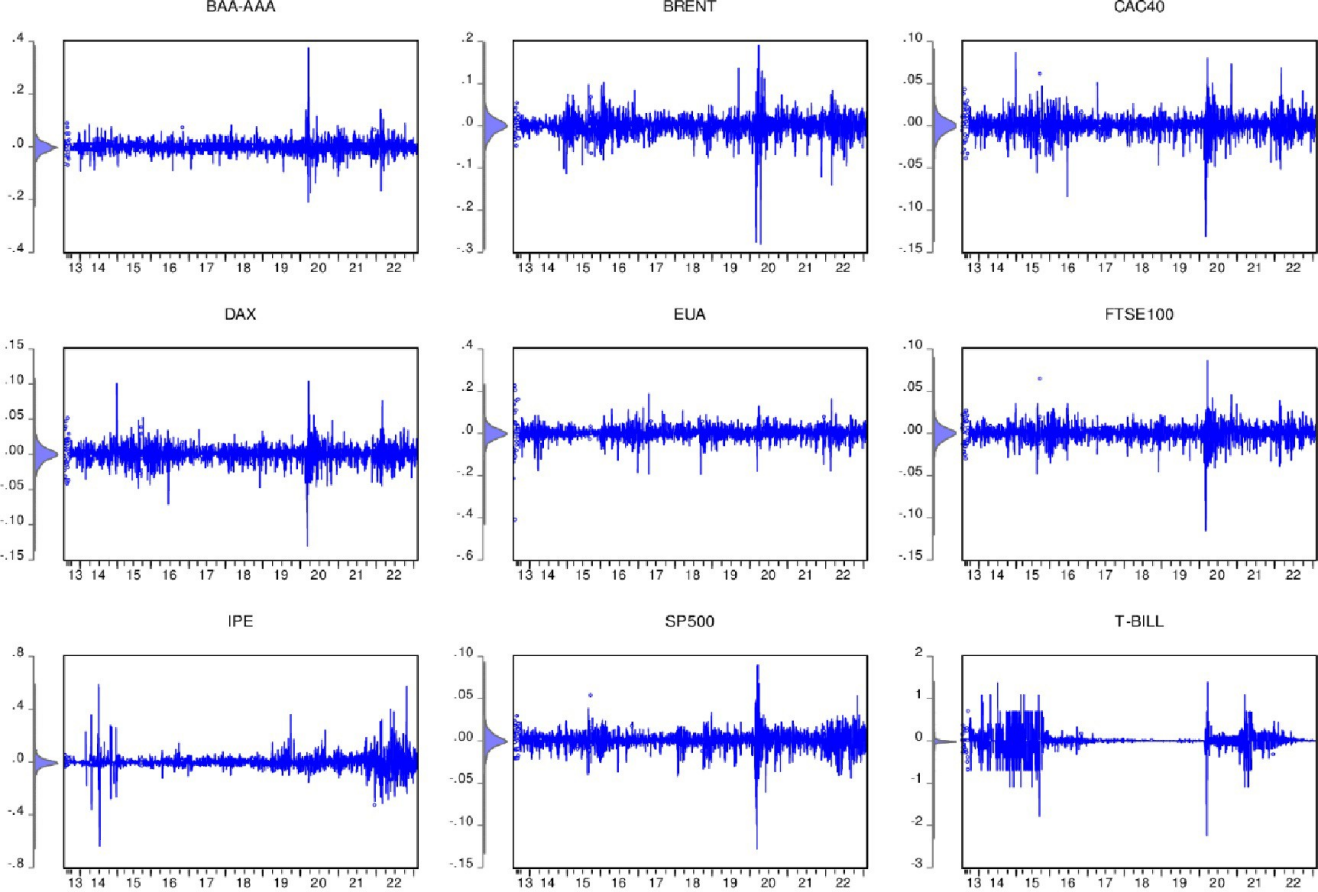
Variable rate of return trend display.

Next, this article uses the formula *R*_*t*_ = *ln*(*P*_*t*_/*P*_*t*-1_) to obtain the logarithmic return sequence, where is *P*_*t*_ the daily closing price and is *P*_*t*_ the market closing price of the previous day. Based on these return data, we explore return spillovers between carbon, fossil energy and financial markets. The specific income trend is as follows in Fig 2:

Fig 2 provides an intuitive visual representation that clearly reveals the significant volatility clustering effects that exist across markets. This volatility aggregation effect usually stems from the close connection and interdependence between markets. For example, when a large fluctuation occurs in one market, this fluctuation may spread among other markets, causing the volatility of other markets to also increase. Furthermore, we can see from the chart that most markets saw significant extreme gains around 2020. This widespread return volatility may stem from the global epidemic that year and the resulting turmoil in global financial markets. Especially for BRENT crude oil returns and S&P SP500 returns, the volatility is more significant. This may be attributed to price fluctuations in the crude oil market as well as large swings in the stock market. The dramatic fluctuations in S& P SP500 earnings may reflect the general stock market environment that year. Due to the economic uncertainty caused by the epidemic, investors may have had excessive panic and excessive optimism about the stock market, which in turn led to the ups and downs of the stock market. The fluctuations in the BRENT crude oil market may be due to the global epidemic having a major impact on the supply and demand relationship in the crude oil market, resulting in violent fluctuations in crude oil prices.

### 4.2 Related tests and descriptive analysis

In addition, relevant statistical analysis of each variable was conducted, as shown in Table 2:

**Table 2:** Descriptive statistics and unit root test.

Statistics	Mean	Maximum	Minimum	Std. Dev.	Jarque-Bera	Prob	ADF (Prob)
**EUA**	0.0012	0.2231	-0.4129	0.03	20718	0.00	0.00
**SP500**	0.0005	0.0897	-0.1277	0.01	22259	0.00	0.00
**T_BILL**	0.0019	1.3863	-2.2513	0.21	27072	0.00	0.00
**CAC40**	0.0003	0.0871	-0.1310	0.01	10078	0.00	0.00
**DAX**	0.0003	0.1041	-0.1305	0.01	9049	0.00	0.00
**FTSE100**	0.0001	0.0867	-0.1151	0.01	14260	0.00	0.00
**BRENT**	-0.0001	0.1908	-0.2798	0.03	23424	0.00	0.00
**IPE**	0.0003	0.5849	-0.6356	0.06	48546	0.00	0.00
**BAA_AAA**	0.0000	0.3759	-0.2095	0.03	61193	0.00	0.00

**Fig 3:**
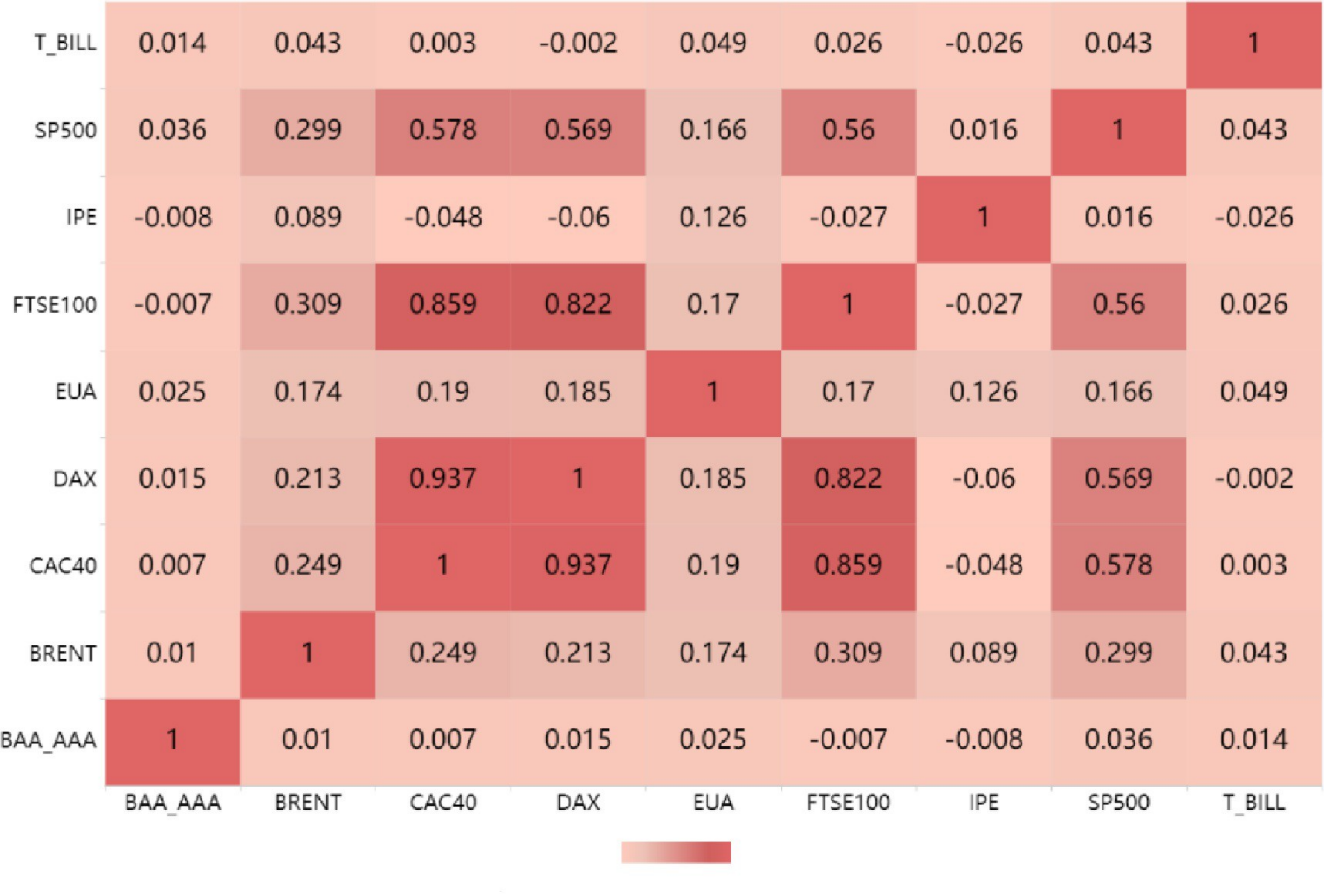
Correlation analysis.

It can be seen from Table 2: The average return rate of B RENT crude oil is negative, while the average return rate of other variables is positive. Among all markets, T -B ill has the largest average return value and standard deviation. Secondly, the EU carbon market (EUA) has the highest rate of return, indicating that the overall carbon market is developing well. From the J -B statistic, the corresponding P values are all 0.00, rejecting the null hypothesis, indicating that all variables do not obey the normal distribution. Finally, the unit root test (ADF test) statistic shows that all-time series are stable at the 1% level. Secondly, the correlation between each market and the EU carbon market is calculated, as shown in Fig 3.

It can be seen from Fig 3 that the correlation between EUA and financial market indicators is relatively high, especially with the S&P 500 and the returns of the three major European stock markets, such as CAC40 and DAX. Secondly, the correlation between the EU carbon market and B RENT returns is also relatively large, indicating that overall, there is a significant correlation between the EU carbon market, the financial market and the energy market.

### 4.3 Overflow network analysis

Next, the spillover index is measured using a rolling window size of 100 days (W) and a forecast horizon of 10 days (H). The overall static overflow index is shown in Table 3:

**Table 3:** Overall inter-market spillover index.

Variable	EUA	SP500	T_BILL	CAC40	DAX	FTSE100	BRENT	IPE	BAA_AAA	From
**EUA**	99.2	0.1	0.0	0.2	0.4	0.0	0.0	0.2	0.0	0.8
**SP500**	2.9	96.5	0.3	0.1	0.0	0.2	0.0	0.0	0.0	3.5
**T_BILL**	0.2	0.1	99.3	0.0	0.0	0.1	0.0	0.0	0.2	0.7
**CAC40**	3.8	35.1	0.1	60.9	0.1	0.0	0.0	0.0	0.0	39.1
**DAX**	3.5	34.2	0.1	49.8	12.1	0.1	0.1	0.0	0.1	87.9
**FTSE100**	3.1	34.0	0.1	37.6	0.3	24.8	0.0	0.0	0.1	75.2
**BRENT**	3.3	7.3	0.3	0.6	0.6	2.9	84.8	0.1	0.1	15.2
**IPE**	1.5	0.4	0.1	0.6	0.3	0.1	0.6	96.4	0.1	3.6
**BAA_AAA**	0.1	0.3	0.3	0.0	0.1	0.2	0.2	0.0	98.7	1.3
**To**	18.3	111.4	1.3	88.9	1.7	3.7	1.0	0.4	0.6	227.3
**To(inclu own)**	117.5	207.9	100.6	149.8	13.8	28.5	85.8	96.8	99.3	25.30%

**Fig 4:**
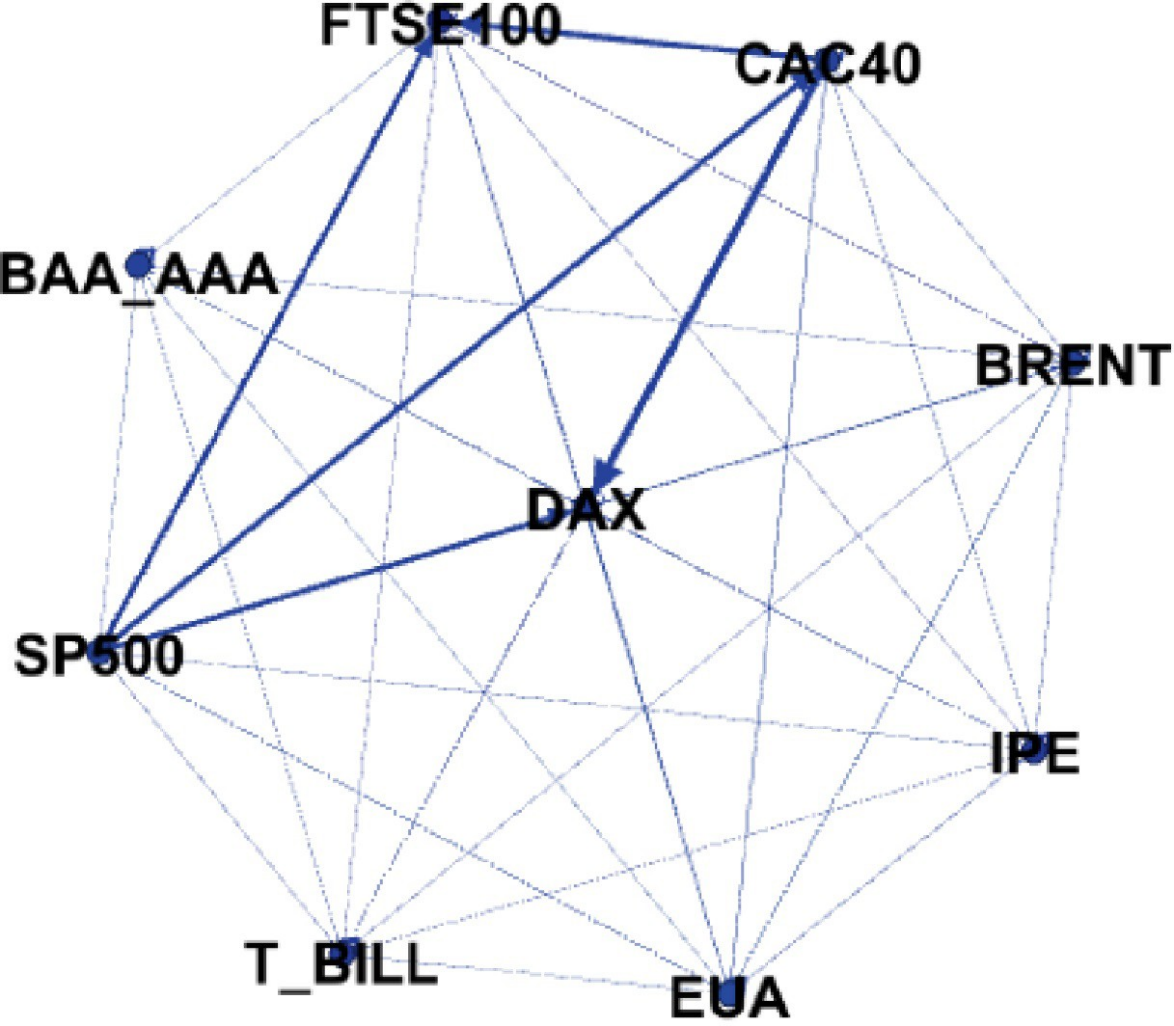
Directed connectivity network between markets.

Overall, the overall spillover index between markets is 25.30%, indicating that there are significant spillover risks between markets. From a diagonal perspective, whether it is financial markets, energy markets or carbon markets, they are mainly affected by their own risk spillovers. Second, looking at each column, for example, looking at the first column, the EUA market spilled out by 18.3% of the risk value. Among them, C AC40 and D AX in the financial market have the greatest impact, and the B RENT crude oil spill risk in the energy market is greater. It shows that when risks occur in the carbon market, they will be mainly transmitted to the European financial market and energy market. In comparison, the EU carbon market has the smallest spillover to the bond market, at 0.1%. In addition, it can be seen that the S&P 500 has strong external spillovers. As a global weather vane, when risks occur in its stock market, it will be transmitted to various markets quickly. In addition, from each row, changes in the EU carbon market are mainly affected by DAX and C AC40, indicating that changes in European market stocks will be transmitted to the EU carbon market. Next, Fig 4 shows the directional connectivity network between markets.

According to the display in Fig 4, we can see that SP500, CAC40 and EUA play the role of net senders of risk spillovers in the system. That is to say, the behavior of these markets in the system will produce more risk spillovers to other markets. In contrast, other markets such as DAX, FTSE100 and HK have become net receivers of risk and are more affected by other markets. It is worth noting that the carbon market has significant spillover effects on DAX and BRENT, indicating that changes in the carbon market may have a significant impact on these two markets. On the other hand, DAX, CAC40 and SP500 will also have a spillover effect on the carbon market, which means that changes in these markets may have an impact on the carbon market, making the carbon market vulnerable to their impact. From these results, we can see that the spillover risk between financial markets is relatively large and there is a significant co-movement effect. This linkage effect may come from the close connection and mutual influence between different markets, such as the cross-border allocation of investment portfolios and the financial settlement of cross-border trade. These factors cause fluctuations in different markets to have a certain chain reaction, so changes in one market may have a significant impact on other markets.

### 4.4 Dynamic overflow index

**Fig 5:**
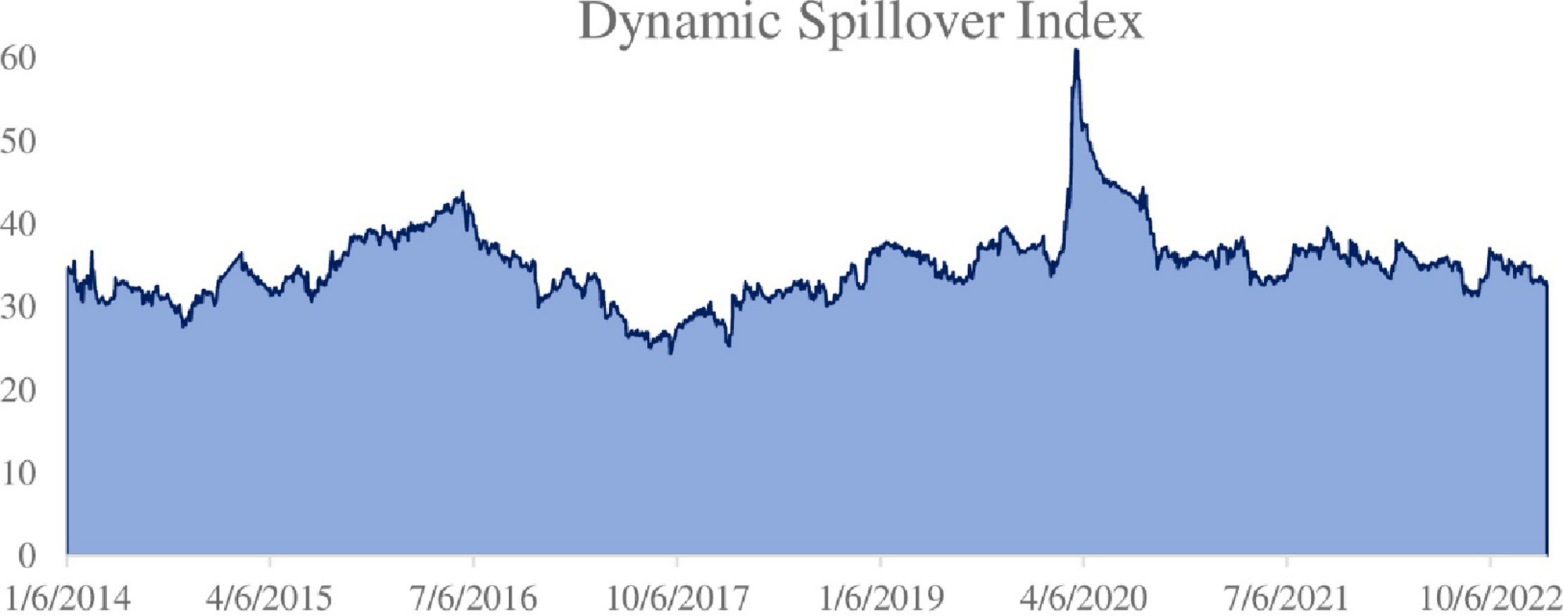
System dynamic overflow index.

Next, the dynamic spillover results during the sample period are presented. As shown in the Fig 5 below, it provides a reference for analyzing the time-varying characteristics of spillover effects.

In the time domain framework of dynamic analysis, the spillover index varies between 24.37% and 61.01%. Various economic and financial uncertainty events are important determinants of increased total spillovers between carbon and financial and energy markets. During the 2020 COVID -19 epidemic, the total spillover rate increased sharply, reaching the highest value at 61.01% when the global COVID-19 epidemic was at its worst (July 2020). and poses huge risks to carbon and energy markets. This further confirms that under deteriorating economic and financial conditions, investors react faster to the spread of negative information, leading to significantly increased spillover effects. This suggests that as the risk event prolongs, the uncertainty caused by the shock gradually intensifies the spillover effect. From the beginning of 2021, as the epidemic control policies of various countries have gradually strengthened, the total spillover effect has declined, and the connection between the carbon market and the energy market has decreased, but it still shows a high level, basically maintaining more than 30%.

### 4.5 Directional spillover index

**Fig 6:**
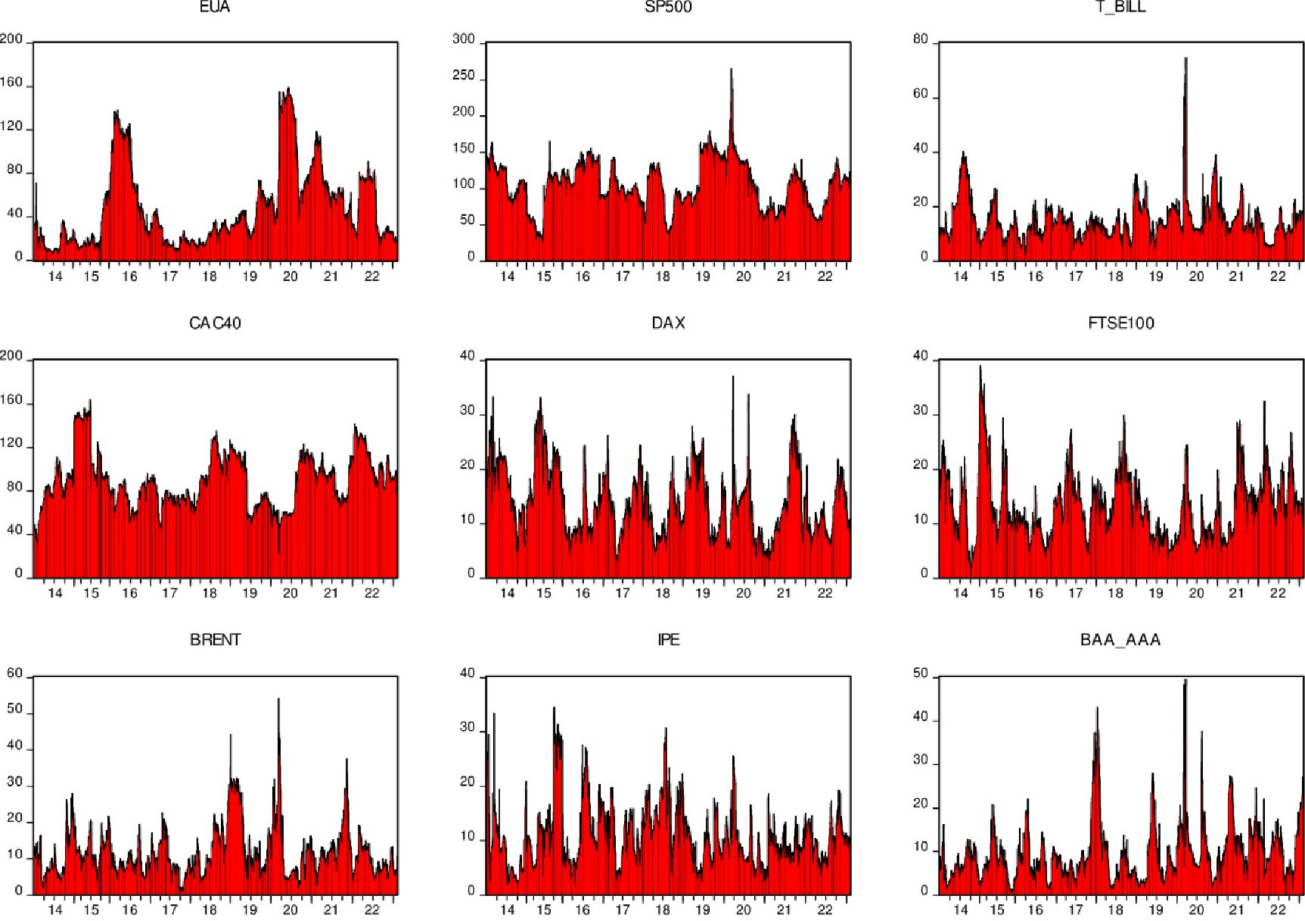
Dynamic time-varying marginal spillover index.

**Fig 7:**
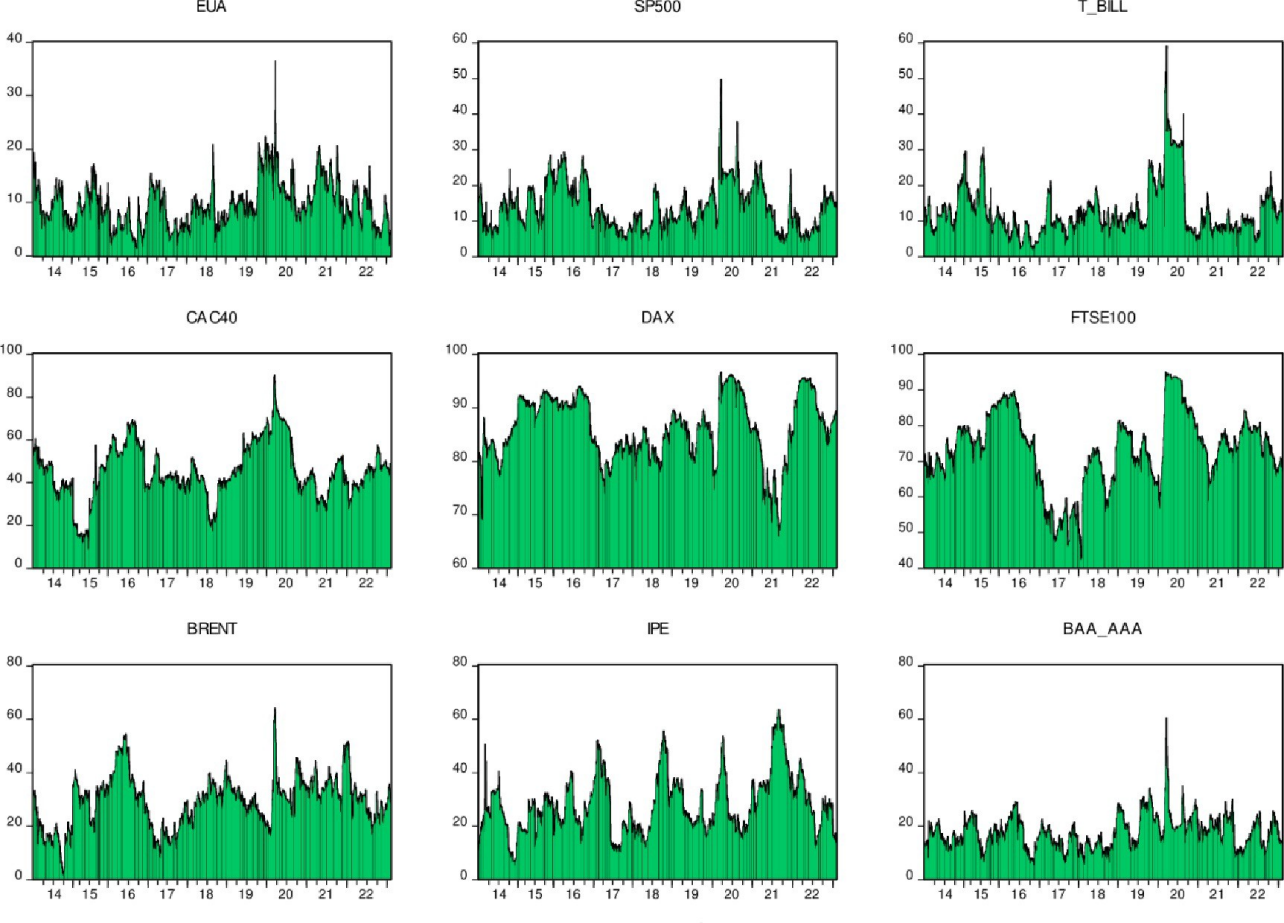
Dynamic time-varying overflow index.

Next, the dynamic risk spillovers between various markets are shown (Fig 6):

From the dynamic spillover index in Fig 6, CAC40, S P500 and EU carbon market E UA have stronger risk spillovers to other markets, but this spillover changes dynamically over time. On average, for the EU carbon market, spillovers peaked between 2015 and 2016, a period when the EU carbon market underwent a series of policy changes, including tighter regulations on the energy sector. Emission limits, expanded market coverage, etc. These changes could lead to increased carbon price volatility, increasing risks in other markets. In addition, the spillover effect of the EU carbon market reached its maximum in 2020. This may be due to the impact of the 2020 epidemic. The epidemic may increase the spillover risk of the EU carbon market in 2020 by affecting economic activities, supply and demand relations, investor sentiment, and policy measures. However, it should be noted that this is only one of the possible factors, and the specific impact is also affected by the complex interaction of other factors and the market. Next, the dynamic risk overflow situation is shown:

According to Fig 7, we can see that the CAC40, DAX and FTSE100 suffered the largest risk spillovers among the nine markets studied. This means that from the overall perspective of these nine markets, these three markets have become net recipients of risk. In financial markets, this risk spillover phenomenon is often caused by the interdependence and linkage between markets. For example, when one market experiences volatility, that volatility may spread across other markets and cause volatility in other markets. It is also worth noting that the carbon market will be most affected by external market spillovers in 2020. This may be related to the occurrence of the new coronavirus pneumonia epidemic. Under the impact of this global epidemic, all markets have been affected to varying degrees, and the carbon market is no exception. Although the volatility of the carbon market may not be as pronounced as other markets, the continued convergence of global macroeconomic, financial and commodity markets make the carbon market inevitably affected by the transmission of fluctuations in other markets. This means that any disturbance in other markets may affect the carbon market, making it vulnerable to impacts from other markets. Next, we further present the dynamic net spillover index. This index can help us better understand the spread and impact of volatility across different markets. By calculating this index, we can more accurately grasp the interrelationships and mechanisms between different markets, thereby better predicting and responding to market risks and fluctuations.

**Fig 8:**
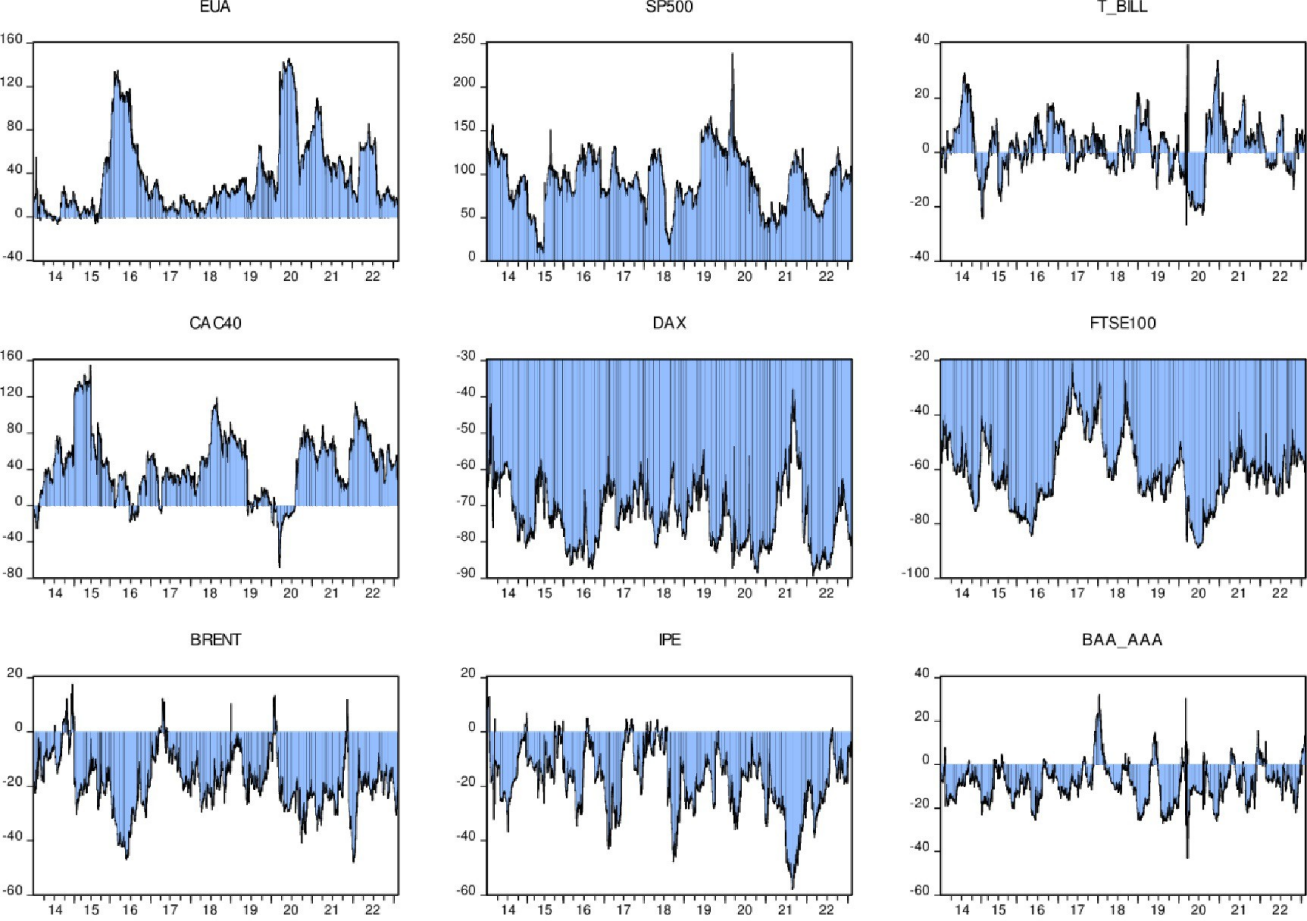
Dynamic time-varying net spillover index.

From Fig 8 we can gain insight into the complexity and sensitivity of financial markets. Both markets, EUA and SP500, have been shown to be risk emitters throughout the study period, which means that risk changes in these two markets will directly affect other markets, highlighting their importance in the global financial system. On the contrary, DAX and FTSE100 behave relatively conservatively. They act more as net spillover recipients of risks, that is, they are more susceptible to other market risks, which further confirms the fragility and sensitivity of financial markets. Especially during the COVID-19 epidemic, we can see a significant increase in the risk spillover effects of EUA and SP500. This may be because during this period, the global supply chain and production network were severely impacted, leading to greater instability in these two markets that were already sensitive to external risks. At the same time, due to the impact of the epidemic on the global economy, a large amount of uncertainty has also emerged in these two markets, further exacerbating risk spillovers. Overall, these results indicate that financial markets are barometers of the global economic environment and are extremely sensitive to changes in the macroeconomic environment in which they operate. When faced with external risks, financial markets can quickly respond to and transmit these risks, thus having a profound impact on the global economy. This also reminds us that as globalization deepens today, we need to pay more attention to the supervision and risk management of financial markets to prevent its fluctuations from causing excessive impact on the global economy.

### 4.6 Pairwise overflow index

Figs 6 and 7 plot dynamic pairwise directional spillovers between carbon and energy markets. Negative spillover values indicate that other markets have net spillovers to the carbon market, and positive values indicate that the carbon market has net spillovers to other markets. Furthermore, this paper finds that the dynamic transmission properties of carbon, financial and energy markets vary across energy types.

**Fig 9:**
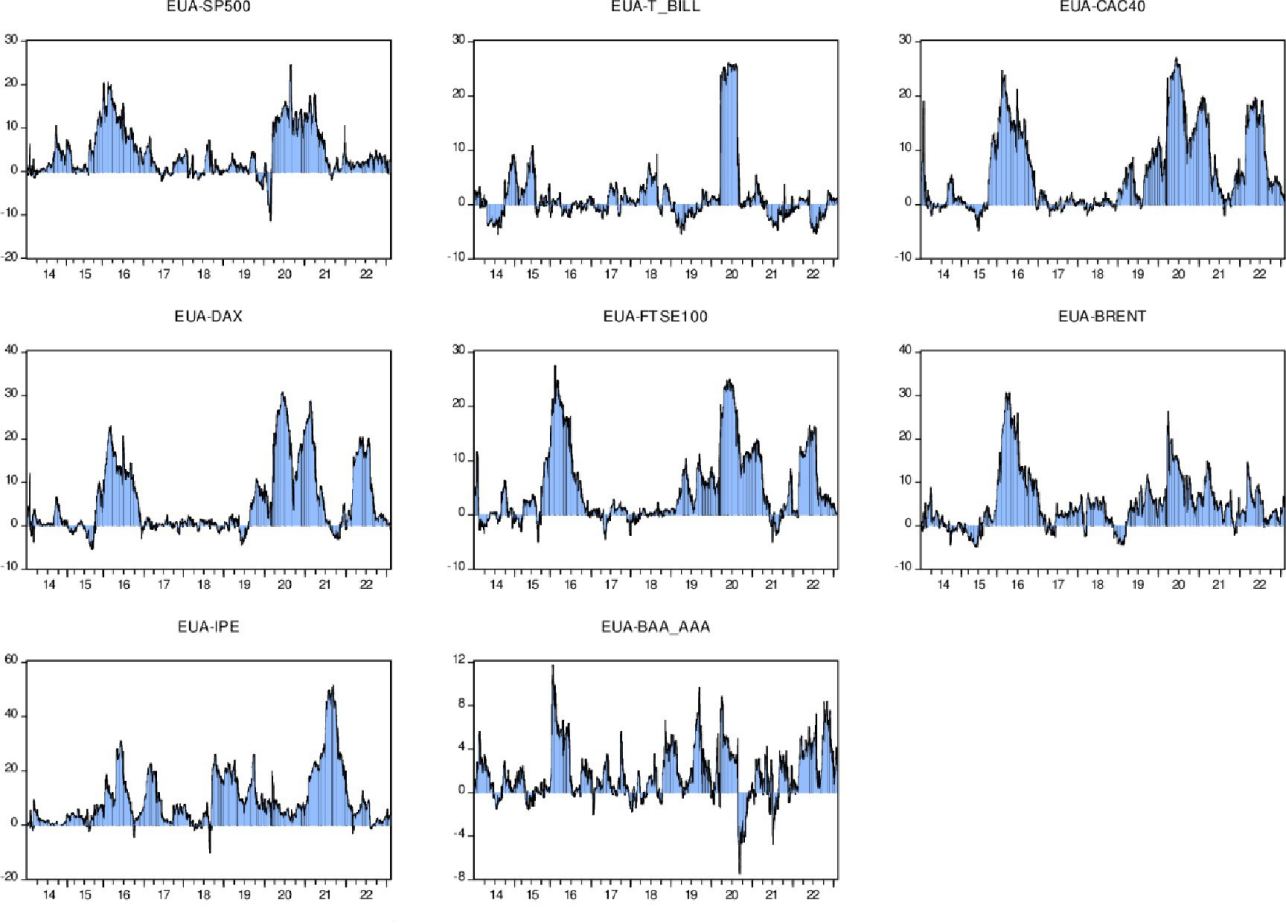
Dynamic pairwise overflow index.

In Fig 9, we observe pairwise spillovers between the EU carbon market (EUA) and eight other markets. This figure clearly shows that the EU carbon market has a significant impact on other markets, and this impact is increasing year by year, forming a powerful spillover effect. This phenomenon fully illustrates the important position of the EU carbon market in today’s global market. The status of the EU carbon market is increasing year by year, and there are many factors behind this trend. First of all, as one of the world’s largest economies, the EU’s policy influence cannot be ignored. The EU carbon market policies and measures can often affect other countries and regions, thereby affecting the global carbon market. Secondly, the EU carbon market has a relatively complete mechanism, strong policy implementation, and high credibility and transparency. This has enabled the EU carbon market to attract a large amount of investment and transactions, further consolidating its dominant position in the global carbon market. The EU carbon market not only affects the financial market, but also profoundly affects the energy market. In terms of financial markets, price fluctuations and policy adjustments in the EU carbon market have a significant impact on global financial investment. For example, the EU carbon market’s emission reduction policies may lead to reduced investment opportunities in certain industries, thereby affecting the flow of global capital. In terms of the energy market, the EU’s carbon emissions trading mechanism has promoted the development of clean energy and has had an important impact on the adjustment of the global energy structure. In addition, the trading mechanism of the EU carbon market has also added a new dimension to the fluctuation of energy prices, which has had a profound impact on the global energy market. In short, we can see from Fig 9 that the EU carbon market has significant spillover effects on other markets, which fully proves its dominant position in the global carbon market. Over time, this spillover effect may further intensify, having a greater impact on global financial and energy markets. Therefore, we must pay close attention to the dynamics of the EU carbon market in order to better understand and respond to its impact on the global economy and energy landscape.

## 5. Conclusion

As climate change becomes a major global issue, recognition of the need for an energy transition is profoundly changing the link between carbon and energy markets. This study combines the DY method to explore the spillover effects between carbon, fossil energy and financial markets. Then, this study introduces the marginal net risk spillover index to analyze the intensity, sources and channels of cross—market risk contagion during the sample period. Several conclusions can be drawn from this.

(1) The spillover effects between carbon markets, financial markets, and energy markets change over time and are strongly influenced by extreme events. During the sample period, the overall spillover index was 25.30%, indicating that the mutual influence and interaction between the three markets were relatively small. However, during the COVID-19, the spillover index of spillover effect increased significantly, reaching more than 60%. This high overflow index may reflect multiple factors. Firstly, the pandemic has had a significant impact on the global economy and financial markets, leading to disruptions in production and operation in many industries, thereby affecting the supply and demand relationship in the carbon and energy markets. In short, the spillover effects between carbon markets, financial markets, and energy markets change over time and are influenced by multiple factors. During the COVID-19, the spillover index of spillover effect rose sharply, which may lead to market volatility and instability. Therefore, for investors, they can adjust their investment by paying attention to changes in the spillover network, especially during the pandemic. Due to the strengthened connectivity of the three markets, when investors have already invested in the carbon market, financial market, and energy market, they can shift to other markets for risk diversification and avoid losses. For the government, it is important to closely monitor the changes in the dynamic spillover index. When the spillover index between the three is high, the carbon market can be regulated by regulating the financial and energy markets. For example, around 2020, T-BILL had a significant spillover effect on the carbon market. Therefore, the government can influence T-BILL by adjusting the money supply or tax revenue, and then transmit it to the carbon market to regulate carbon market prices.
(2) There are significant differences in the spillover effects of different financial, energy, and carbon markets, which may be related to the characteristics and development stages of different markets. This may be related to the characteristics and development stages of different markets. Therefore, when formulating carbon neutrality related policies, it is necessary to understand the spillover effects between different markets in order to better predict market dynamics and formulate appropriate policies. The carbon market is a relatively emerging market, with relatively active price fluctuations and trading activities, making it more susceptible to the influence of other markets. Specifically, the carbon market is susceptible to shocks from markets such as Brent crude oil, CAC40, and DAX. This may be related to the high correlation between these markets, as they are both important energy and financial markets and closely related to the global economic and trade situation. In the long run, the carbon market may be a net spillover emitter, transmitting spillover effects to other markets such as the bond market and natural gas market. Therefore, it is necessary to promote the mature development of the carbon market in order to better play its role in controlling carbon emissions. The government can take measures to increase investors’ confidence and participation in the carbon market, such as providing policy and financial support, establishing a sound regulatory system, etc. In addition, governments and financial institutions need to closely monitor the spillover effects between different markets, strengthen macro prudential management and risk control, to ensure economic and financial stability. In addition, it is necessary to strengthen the establishment and improvement of cross market supervision and cooperation mechanisms, in order to better serve the sustainable development of the economy and society.
(3) During the COVID-19, the global market suffered an unprecedented impact, and the spillover effect between the carbon market, the fossil energy market and the financial market increased significantly. In this crisis, the carbon market has become a major risk generator, indicating their increasing influence. Firstly, due to the serious impact of the epidemic on the global economy, governments around the world have taken various measures to control the spread of the epidemic, which has led to changes in the global energy consumption structure. As people travel and gather less, commercial and industrial activities decrease, and the demand for energy also correspondingly decreases. In this situation, the fossil energy market has been severely affected, with prices of energy such as oil, natural gas, and coal plummeting significantly. However, the carbon market has emerged in this crisis. The carbon market is a financial market that trades carbon emission rights. It is market-oriented and promotes enterprises to reduce carbon emissions and achieve environmental protection goals. Due to the high liquidity and transparency of the carbon market, it can attract a large amount of funds and investors’ attention. In this context, the carbon market has gradually become the main risk transmitter. Due to the close correlation between carbon market price fluctuations and the fossil energy market, carbon market price fluctuations can also have an impact on the fossil energy market. In addition, price fluctuations in the carbon market may also have an impact on the financial market, as price fluctuations in the carbon market may affect corporate profits and cash flows, thereby affecting stock and bond prices. Therefore, the government should strengthen regulation, establish stricter carbon emission standards, and promote enterprises to reduce carbon emissions. At the same time, the government should also increase investment in the carbon market and encourage more companies and investors to participate in carbon market trading. In addition, the coordinated development of the fossil energy market and the carbon market should be promoted. The government should formulate more flexible energy policies, encourage enterprises to transform their energy consumption structure, increase support for clean energy, and promote the coordinated development of the fossil energy market and carbon market. Overall, the research in this article indicates that the fossil energy market has been severely affected by the pandemic, while the influence of the carbon market is gradually increasing. The government can take measures to promote the transformation of energy structure, increase support for clean energy, encourage enterprises to change their energy consumption structure, reduce carbon emissions, and achieve carbon peaking and carbon neutrality faster and better.

## Supporting information

S1 Data (XLSX)

## References

[1] Ding Q, Huang J, Zhang H. Time-frequency spillovers among carbon, fossil energy and clean energy markets: The effects of attention to climate change. Int Rev Financ Anal. 2022;83: 102222. doi: 10.1016/j.irfa.2022.102222

[2] Zhang H, Zhang Y, Gao W, Li Y. Extreme quantile spillovers and drivers among clean energy, electricity and energy metals markets. Int Rev Financ Anal. 2023;86: 102474. doi: 10.1016/j.irfa.2022.102474

[3] Alsalman Z, Herrera AM, Rangaraju SK. Oil news shocks and the U.S. stock market. Energy Econ. 2023;126: 106891. doi: 10.1016/j.eneco.2023.106891

[4] Duan K, Ren X, Shi Y, Mishra T, Yan C. The marginal impacts of energy prices on carbon price variations: Evidence from a quantile-on-quantile approach. Energy Econ. 2021;95: 105131. doi: 10.1016/j.eneco.2021.105131

[5] Batten JA, Maddox GE, Young MR. Does weather, or energy prices, affect carbon prices? Energy Econ. 2021;96: 105016. doi: 10.1016/j.eneco.2020.105016

[6] Zhao LT, Miao J, Qu S, Chen XH. A multi-factor integrated model for carbon price forecasting: Market interaction promoting carbon emission reduction. Sci Total Environ. 2021;796: 149110. doi: 10.1016/j.scitotenv.2021.149110 34328877

[7] Wang Q, Fan Z. Green finance and investment behavior of renewable energy enterprises: A case study of China. Int Rev Financ Anal. 2023;87: 102564. doi: 10.1016/j.irfa.2023.102564

[8] Dai Z, Zhu H. Dynamic risk spillover among crude oil, economic policy uncertainty and Chinese financial sectors. Int Rev Econ Financ. 2023;83: 421–450. doi: 10.1016/j.iref.2022.09.005

[9] Dichtl H, Drobetz W, Otto T. Forecasting Stock Market Crashes via Machine Learning. J Financ Stab. 2023;65: 101099. doi: 10.1016/j.jfs.2022.101099

[10] Bao Z, Huang D. Shadow Banking in a Crisis: Evidence from Fintech During COVID-19. J Financ Quant Anal. 2021/07/16. 2021;56: 2320–2355. doi: 10.1017/S0022109021000430

[11] Siddique MA, Nobanee H, Karim S, Naz F. Do green financial markets offset the risk of cryptocurrencies and carbon markets? Int Rev Econ Financ. 2023;86: 822–833. doi: 10.1016/j.iref.2023.04.005

[12] Qiao S, Dang YJ, Ren ZY, Zhang KQ. The dynamic spillovers among carbon, fossil energy and electricity markets based on a TVP-VAR-SV method. Energy. 2023;266: 126344. doi: 10.1016/j.energy.2022.126344

[13] Liu J, Mao W, Qiao X. Dynamic and asymmetric effects between carbon emission trading, financial uncertainties, and Chinese industry stocks: Evidence from quantile-on-quantile and causality-in-quantiles analysis. North Am J Econ Financ. 2023;65: 101883. doi: 10.1016/j.najef.2023.101883

[14] Liu J, Hu Y, Yan LZ, Chang CP. Volatility spillover and hedging strategies between the European carbon emissions and energy markets. Energy Strateg Rev. 2023;46: 101058. doi: 10.1016/j.esr.2023.101058

[15] Wen X, Bouri E, Roubaud D. Can energy commodity futures add to the value of carbon assets? Econ Model. 2017;62: 194–206. doi: 10.1016/j.econmod.2016.12.022

[16] Ma Y, Wang L, Zhang T. Research on the dynamic linkage among the carbon emission trading, energy and capital markets. J Clean Prod. 2020;272: 122717. doi: 10.1016/j.jclepro.2020.122717

[17] Jiao L, Liao Y, Zhou Q. Predicting carbon market risk using information from macroeconomic fundamentals. Energy Econ. 2018;73: 212–227. doi: 10.1016/j.eneco.2018.05.008

[18] Jiménez-Rodríguez R. What happens to the relationship between EU allowances prices and stock market indices in Europe? Energy Econ. 2019;81: 13–24. doi: 10.1016/j.eneco.2019.03.002

[19] Diebold FX, Yilmaz K. Better to give than to receive: Predictive directional measurement of volatility spillovers. Int J Forecast. 2012;28: 57–66. doi: 10.1016/j.ijforecast.2011.02.006

[20] Chen M, Li N, Zheng L, Huang D, Wu B. Dynamic correlation of market connectivity, risk spillover and abnormal volatility in stock price. Phys A Stat Mech its Appl. 2022;587: 126506. doi: 10.1016/j.physa.2021.126506

[21] Chen M, Wang Y, Wu B, Huang D. Dynamic analyses of contagion risk and module evolution on the sse a-shares market based on minimum information entropy. Entropy. 2021;23. doi: 10.3390/e23040434 33917234 PMC8068080

[22] Wu B, Huang D, Chen M. Estimating contagion mechanism in global equity market with time-zone effect. Financ Manag. 2023;52: 543–572. doi: 10.1111/fima.12430

[23] Balcilar M, Demirer R, Hammoudeh S, Nguyen DK. Risk spillovers across the energy and carbon markets and hedging strategies for carbon risk. Energy Econ. 2016;54: 159–172. doi: 10.1016/j.eneco.2015.11.003

[24] Ali M, Tursoy T, Samour A, Moyo D, Konneh A. Testing the impact of the gold price, oil price, and renewable energy on carbon emissions in South Africa: Novel evidence from bootstrap ARDL and NARDL approaches. Resour Policy. 2022;79: 102984. doi: 10.1016/j.resourpol.2022.102984

[25] Yuan N, Yang L. Asymmetric risk spillover between financial market uncertainty and the carbon market: A GAS–DCS–copula approach. J Clean Prod. 2020;259: 120750. doi: 10.1016/j.jclepro.2020.120750

[26] Gao Q, Zeng H, Sun G, Li J. Extreme risk spillover from uncertainty to carbon markets in China and the EU—A time varying copula approach. J Environ Manage. 2023;326: 116634. doi: 10.1016/j.jenvman.2022.116634 36423437

[27] Tan XP, Wang XY. Dependence changes between the carbon price and its fundamentals: A quantile regression approach. Appl Energy. 2017;190: 306–325. doi: 10.1016/j.apenergy.2016.12.116

[28] Yang T, Dong Q, Du M, Du Q. Geopolitical risks, oil price shocks and inflation: Evidence from a TVP–SV–VAR approach. Energy Econ. 2023;127: 107099. doi: 10.1016/j.eneco.2023.107099

[29] Liu R, He L, Xia Y, Fu Y, Chen L. Research on the time-varying effects among green finance markets in China: A fresh evidence from multi-frequency scale perspective. North Am J Econ Financ. 2023;66: 101914. doi: 10.1016/j.najef.2023.101914

[30] Yang MY, Chen Z, Liang Z, Li SP. Dynamic and asymmetric connectedness in the global “Carbon-Energy-Stock” system under shocks from exogenous events. J Commod Mark. 2023;32: 100366. doi: 10.1016/j.jcomm.2023.100366

[31] Tan X, Sirichand K, Vivian A, Wang X. How connected is the carbon market to energy and financial markets? A systematic analysis of spillovers and dynamics. Energy Econ. 2020;90: 104870. doi: 10.1016/j.eneco.2020.104870

[32] Matinmikko-Blue M, Yrjölä S, Ahokangas P. Multi-perspective approach for developing sustainable 6G mobile communications. Telecomm Policy. 2023. doi: 10.1016/j.telpol.2023.102640

[33] Bao Z, Huang D. Can Artificial Intelligence Improve Gender Equality? Evidence from a Natural Experiment. SSRN Electron J. 2022. doi: 10.2139/ssrn.4202239

[34] Bao Z, Abbink K, Agarwal S, Berg T, Bohren A, Chen C, et al. Gender Differences in Reactions to Enforcement Mechanisms: A Large-Scale Natural Field Experiment * First draft: July 2020 This draft: October 2023 Gender Differences in Reactions to Enforcement Mechanisms: A Large-Scale Natural Field Experiment. 2023.

[35] Bao Z, Huang D. Reform scientific elections to improve gender equality. Nat Hum Behav. 2022;6: 478–479. doi: 10.1038/s41562-022-01322-w 35273356

